# Psychiatric risk factors in Formula One and the importance of integrating mental health into driver science

**DOI:** 10.3389/fspor.2024.1480574

**Published:** 2024-10-10

**Authors:** Jill Colangelo, Alexander Smith, Nicky Keay, Ana Buadze, Michael Liebrenz

**Affiliations:** ^1^ Department of Forensic Psychiatry, University of Bern, Bern, Switzerland; ^2^ Honorary Clinical Lecturer, University College London, London, United Kingdom; ^3^ Department of Psychiatry, Psychotherapy and Psychosomatics, Psychiatric Hospital, University of Zurich, Zurich, Switzerland

**Keywords:** mental health, formula 1, motor sports, sports psychiatry, driver science, driver athlete

## Abstract

Formula One (F1) racing has recently grown in popularity, extending well beyond its traditional European roots. However, there has been a paucity of scholarly research dedicated to the health of drivers and even less discussion of the prevalence of psychiatric symptoms, risk factors, and types of psychopathology in F1. This is notable given advancements in sports psychiatry and evidence of psychiatric disorders emerging across other sports. Accordingly, this perspective paper details the physiological conditions in F1 and the socioenvironmental pressures that a driver may encounter during their career, including heat stress, weight restrictions, harassment, and other factors. These extreme physiological and psychological stressors, both in racing and non-racing environments, alongside sport-specific psychosocial pressures, may cause HPA axis dysregulation and other issues in drivers, heightening vulnerabilities for mental health concerns. Additionally, F1 is still affected by stigmatizing attitudes and regressive sociocultural norms, which could inhibit progress toward promoting sustainable wellbeing. Consequently*,* drivers may be at risk for mental disorder and a decrease in overall health and wellbeing. Against this background, we thereby recommend mental health programs and regulatory actions that could better address these challenges and promote mental wellbeing across F1.

## 1 Introduction

Recently, stimulated by commercial rights changes and the influence of Liberty Media, Formula One (F1) racing has garnered wider international interest, with concomitant calls for more thorough scientific research into the sport (1–3). Yet, despite this burgeoning popularity, there has been limited academic attention to the mental health and wellbeing of F1 athletes throughout the history of motor racing. For the purposes of this paper, the definition of wellbeing in a sporting context echoes prior classifications of a multifactorial model, including physical health, strong immunity, energy, quality sleep, coping skills, self-regulation, and satisfactory relationships (4). Under this framework, athlete wellbeing encompasses physical and mental health, as well as a sense of thriving both during one's career and after its conclusion (4).

To the authors’ knowledge, no studies have investigated the prevalence and types of psychiatric symptoms amongst drivers, meaning they have not received the same level of discussion in sports psychiatry and sports medicine as other elite athletes. This is a significant knowledge gap given numerous accounts from drivers describing their experiences with depression, anxiety, and eating disorders (5–10) and reports of them seeking mental health support. For example, 1996 Champion Damon Hill reflected: “I was worthless and not going to make it. It got so bad I went to the manager and said I needed a break and I couldn’t cope” (9). Elsewhere, seven-time champion Lewis Hamilton noted: “I have struggled mentally and emotionally for a long time, to keep going is a constant effort” (10).

The physiological demands of F1 may be uniquely intense, with drivers subjected to extreme environmental conditions. Additionally, socioenvironmental pressures, exogenous dynamics, psychosocial stressors, and stigmatizing attitudes may adversely affect mental health outcomes. The challenges of F1 have long been recognized, though specific terminology and understanding has evolved over decades. Early assessments of motorsport tended to erroneously underestimate its physical and psychological demands (1, 11, 12). This may be expected given the traditional frameworks surrounding F1, where injury risks were both real and anticipated, and could underpin the regressive view drivers of as “gladiators” rather than athletes (13). For example, in the 1970s, the media often highlighted James Hunt's charismatic persona and reckless behavior, somewhat overshadowing the mental demands and physical conditioning required at the elite-level.

Notably, prior research involving motorsport drivers explored topics like crashes and facial injury but did not seem to address the physical and physiological exertions of drivers until the late 1990s (14–17). It is possible that focusing on the driver's personality and character as criteria for success undermined the importance of studying the unique skill and training necessary to compete in F1; certain media narratives may also have shaped regressive perceptions of athletic identity (18, 19). Nevertheless, recent work has highlighted the many physical and psychological challenges athletes face as well as the importance of considering overall safety and wellbeing (2, 20).

Consequently, to draw attention to other ongoing issues in the sport, we present an overview of the psychological and psychosocial stressors in F1, informed by the existing evidence-base (albeit limited) and additional sources. Specifically, this includes physiological stress, sleep deprivation, exercise routines, weight pressures, fatality risks, stereotypes, and gender issues, amongst others. Similar risk factors for mental illness have been discussed in other sports that may have a larger body of supporting literature than F1, alongside those where evidence is emerging, and where organizational structures are less well-developed, such as in ultra-endurance sport (21–23).

In established disciplines, governing bodies such as the International Olympic Committee (IOC) and national federations have created consensus statements to formally address mental health concerns and enhance athlete wellbeing (24). As such, diagnostic and therapeutic plans, as well as support systems can and have been implemented and adapted (25). In short, though psychiatric disorders have been overlooked and stigmatized in sporting contexts for a considerable time, it has become increasingly recognized that athletic success has links with mental wellbeing (24).

F1 has long been characterized by innovation and technological progress, particularly within vehicle designs. Accordingly, to keep pace with advancements elsewhere and better promote health transparency and sustainable wellbeing as defined above, we believe it is imperative to integrate mental health into driver science and larger discussions of psychopathology in elite sports. This is necessary to support the mental health of drivers across the lifespan in ways that would be complementary to the culture of F1 and its legacy. To that end, we propose several recommendations for drivers, teams, the governing body, the Fédération Internationale de l'Automobile (FIA), and additional stakeholders.

## 2 Physiological contributions to psychiatric stress

**Figure 1:**
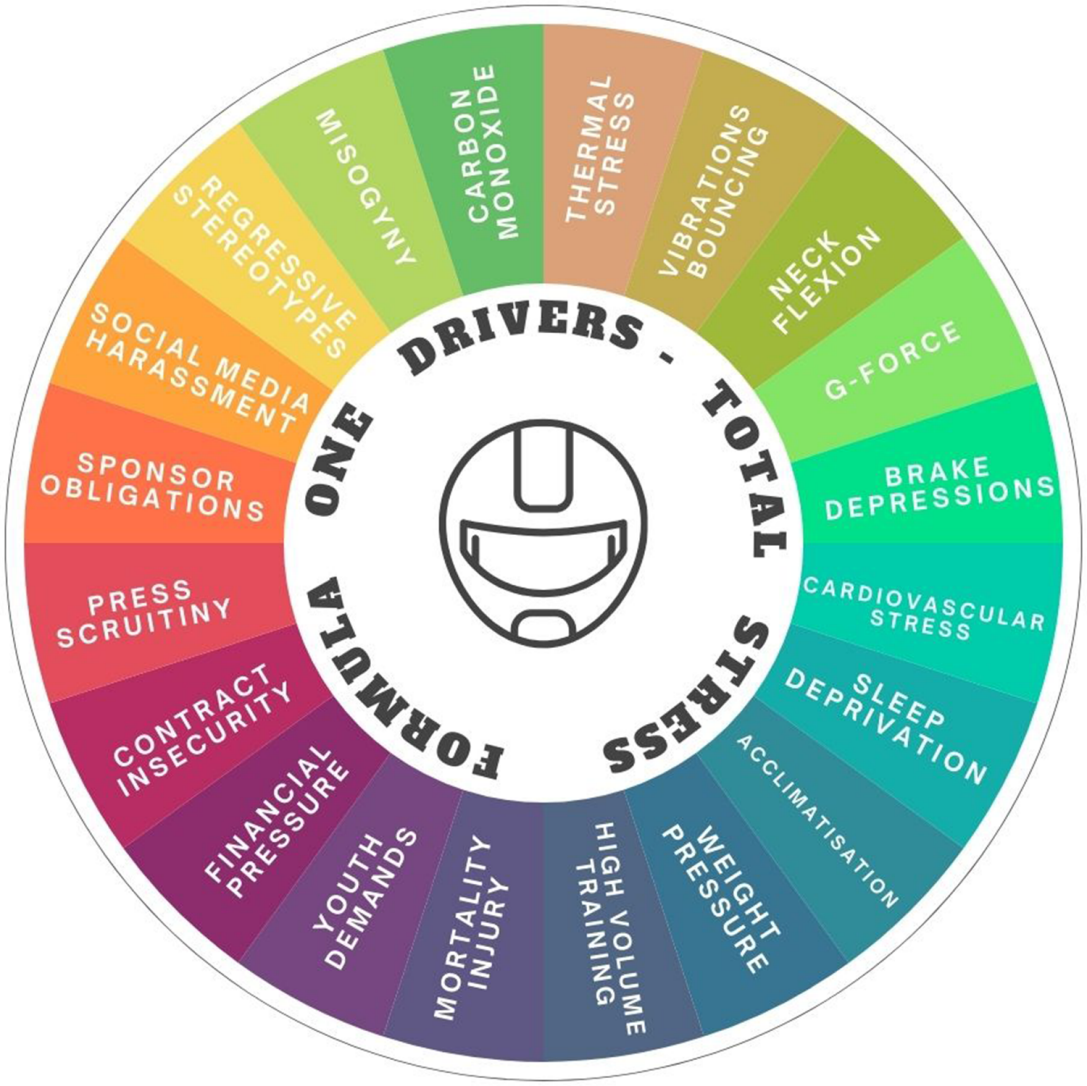
Physiological, psychosocial, and environmental risk factors in F1.

With its immersive and demanding nature, F1 encompasses similar dynamics to other sports that may affect the mental health of competitors, as highlighted in Figure 1.

Specifically, drivers often endure extreme physiological factors in training and racing (20, 26). Like other high-intensity sports, stress from these conditions could activate the hypothalamus-pituitary-adrenal (HPA) axis, which may lead to the release of additional stress hormones, affecting normal neurotransmitter activity with psychiatric implications (27); for example, HPA axis disruption can alter diurnal rhythm and sleep patterns (28). Moreover, such situations can be associated with depression, anxiety, and other disorders, as identified in different scenarios and contexts (29–31). It is therefore important to highlight previous research underlining these physiological demands (e.g. (2, 16, 32), as this could detrimentally affect drivers’ mental health. Further, athletes who enter F1 with pre-existing mental disorders like depression, anxiety, bipolar disorder, etc. and/or who have experienced critical life events may already have an activated HPA axis and resulting higher stress hormones at baseline, thus heightening their vulnerabilities (33).

### 2.1 Physiological stress in training/racing

Though a certain amount of stress is normal and may even be beneficial for training adaptations, the conditions faced by F1 drivers can escalate stress to levels that entail additional risks for HPA axis dysregulation. Notably, drivers may experience extreme thermal stress owing to their fireproof suit (a safety prerequisite), combined with intense intra-vehicle heat (2). Heat stress has been highlighted as a significant problem, especially for hot-climate races, possibly leading to sickness and dehydration (34). Furthermore, intra-vehicle vibrations can increase mental fatigue, provoking variations in cardiac output, and a driver's neck can be induced to violent flexion in turns, braking, and contact with competing vehicles, at times reaching the diagnostic threshold for whiplash [3, 35]. In turn, these conditions have been linked to neurological conditions, sleep disturbances, depression, and other psychiatric disorders [36, 37]. Likewise, *G*-force is a constant stress, as drivers are subject to up to 5 *g* around corners and the head and neck must resist *g* forces while wearing a helmet weighing >6 kg [2, 38].

Resultantly, the effects of intra-race physiological output on the welfare of F1 drivers warrants extensive attention, as these typically differ from practice-run conditions and could have injurious short- and long-term consequences (39). Notably, repetitive depression of the brake requires ≤140 kg of pressure (40). It is likely that the cardiovascular system of drivers faces significant stress as the body manages heat, *g* force, and oxygen consumption, with racers regularly maintaining an average 90% of maximum heart rate in a 90-minute race (2, 41).

### 2.2 Sleep deprivation, exercise routines, and weight pressures

Together with the dynamics described, drivers could experience sleep deprivation owing to their competitive routines (36, 42). Disturbed sleep patterns can dysregulate hormonal rhythms and metabolism, creating a vicious circle of increasing stressor load and negative adaptive responses (43). Characteristically, demanding schedules leave little time for drivers to acclimate, rest, and recover across time zones, and in intra-race environments, driver fatigue could have wide-ranging safety consequences (3, 44).

Prior to 2018 regulation changes, drivers were required to maintain a very low and possibly harmful body weight for performance; several drivers identified difficulties with this, heavily restricting calorie intake and some even requiring hospitalization (45). Specifically, the current driver, Valtteri Bottas, acknowledged these patterns and discussed his experiences with eating disorder symptoms, such as over-exercising, food restriction, and periods of illness (7, 46). Today, drivers still need to adhere to a specific body weight, which, including their seat, must be ≤80 kg. By increasing exercise and decreasing caloric intake and adequate recovery, coupled with the need to maintain lower body weight for performance, drivers may encounter distinctive vulnerabilities. This includes risks for overreaching, Overtraining Syndrome (OTS), eating disorder, or complications associated with Low Energy Availability (LEA), like relative energy deficiency in sport (REDs) with sustained LEA (47, 48). Notably, LEA is associated with myriad health concerns, including hormonal disturbances, stress fractures, depression, and anxiety (47).

## 3 Psychological stressors

Various psychosocial stressors can arise from public-facing events, through to competition, and even possible fear of injury or critical accidents (49). Notably, drivers often experience fatigue due to travel obligations (Figure 1) and must navigate an elite-level environment that has historically been shaped by stereotypes and stigma (50, 51).

### 3.1 Socioenvironmental and psychosocial concerns

Throughout their racing career, F1 drivers may need to fulfill external obligations that invoke stressors. Over a typical Grand Prix weekend, drivers recurrently participate in numerous media engagements, briefings, and obligatory meet-and-greet events for sponsors, alongside training and practice runs (52). Media engagements may incur challenges, even placing stringent expectations on drivers’ public behaviors (53). In this context, the difficulty in shifting focus from engaging socially to race preparation has been emphasized (54). Correspondingly, drivers may have limited time to transition between disparate responsibilities, such as press calls and entering the cockpit, in turn compounding fatigue levels (52, 54).

Recent efforts to bolster race popularity has inadvertently heightened social media scrutiny towards drivers. Occasionally, this has led to harassment and death threats (55, 56), which in other sports has created significant personal difficulties for athletes (57). Likewise, news outlets can be critical of drivers, at times influencing their career outcomes (58). Nevertheless, drivers are not permitted to withdraw from public engagements, as their cultivated persona is an integral part of team popularity, sponsorships, and overall celebrity across F1 (59). For other elite athletes competing in different sports, the pressures and demands of such interactions have been identified as salient determinants of lower mental wellbeing (60).

Moreover, during the so-called “silly season” in F1, contract negotiations can be dramatic and play out publicly, eliciting speculation, rumors, and vocational insecurities (61, 62). Especially during the off-season drivers can face constant pressure about potentially losing their seats for the upcoming race calendar, exemplifying the longstanding notion that: “you are only as good as your last race”. This period has historically been characterized by logistical and practical challenges, with even the best drivers uncertain about career trajectories (63). Further, as drivers can be replaced both in between and mid-season, many have described feelings of anxiety owing to the pressures of maintaining ranking among their own teams and across rival teams (64–66).

Akin to other sporting disciplines, F1 careers can start at an early age; drivers can commence karting as young as three years old, involving considerable time commitments from families and stakeholders (67, 68). Notably, young drivers from South America or Japan can begin racing in European feeder programs and this separation from home and the immersion in highly competitive environments could pose psychological challenges. Analogously, financial investments in a young driver may exceed one million pounds to reach F4, rising to as much as eight million pounds through the feeder programs; inevitably, this can increase performance-based and socioenvironmental pressures on the athlete (69, 70). Some drivers may have been pushed to participate by family members and subsequently encountered stressful situations (71). While athletic participation affords children benefits, intense competition and pressures at a young age may induce stressors or exacerbate susceptibility for later psychiatric conditions (72, 73), suggesting a need for more robust safeguarding measures and regulatory oversight.

It is clear that due to the challenges inherent in the journey to become an F1 driver, a significant amount of intrinsic passion is required to perform and persevere in the sport. Such intense desire could lead to maladaptive behaviors or burnout, and it is possible that mental health struggles may arise from the inability of athletes to manage their achievement needs or “fear of failure” (74, 75). These concerns could be present in young drivers and result in frustration, anxiety, and difficulties with perfectionism throughout their career (76, 77). Similarly, highly motivated athletes may face additional mental health concerns when they end their careers in F1 (78), impinging upon the definitions of sustainable wellbeing outlined above (4). Evidence from other sports indicates that elite athletes are often challenged in retirement as they seek to reconcile their identity independent of the sport (79), which may be equally relevant for F1 drivers. Likewise, those who leave their sport with injuries or chronic pain may be at additional risk for depression and other mental health concerns (80, 81); again, these could be pertinent issues in F1 given its risks for physical injury (20).

### 3.2 Fatality risks and regressive sociocultural constructs

Within F1, injury and morality risks can cause psychological stress (49, 82). Historically, F1 was marked by higher rates of critical incidents than today's races. For instance, the 1970s was a particularly perilous era, where it was not uncommon for multiple fatalities to occur in a single season. Since then, the FIA have implemented numerous positive changes designed to improve safety (83). Recent vehicle modifications have introduced the so-called “Halo” crash protection system in 2018 and a push for stronger roll hoops (84, 85). Despite these advancements, the possibility of serious injuries remain; these episodes can still be catastrophic for drivers, teams, and their families (86).

Nevertheless, racing policies and marketing priorities change continuously and while aimed at boosting the sport, may not always result in better driver welfare. Following what was considered to be a less-exciting racing atmosphere, 2022 saw sweeping rule changes (87). These prompted comprehensive vehicle redesigns, rendering it possible for cars to get closer and allow for more overtaking (88, 89). Moreover, the Grand Prix series management of F1 also expressed interest in monitoring high, intra-race driver heart rates in 2021 (90), but it is unclear if this was for scientific reasons or solely for entertainment purposes.

In the authors’ opinion, these developments could have been influenced by regressive sociocultural paradigms equating F1 with entertainment rather than sport, potentially misidentifying drivers as performers rather than true athletes (91). Previously, the President and CEO of the F1 Group, commented on the intentions behind such actions: “It will create all this drama. I’m pretty sure that most of the drivers will love it because they are fighters. Like in the colosseum in the old days” (92). Yet gladiators were entertainers and were considered exploitable and expendable. Even recently, this issue arose again during an excessively hot competition in Qatar in October, 2023 that was supported and even encouraged by certain media outlets, though it caused illness amongst several athletes (93). Unfortunately, there has been a history of referencing gladiator comparisons in sports, particularly in those that risk bodily harm (94, 95). Albeit hyperbolic, commentary that portrays F1 drivers in this way likely diminishes mental and physical health concerns around crashes and may even amplify dehumanizing messaging or regressive perceptions of athletic identity (93).

Ignoring dangers or manipulating aspects of the racing environment to increase excitement is not new in F1 and unfortunately, was traditionally common across motorsports (13). Previously, safety was not a prominent consideration, with drivers celebrated for their bravery irrespective of danger, though certain commentators have questioned the relative appeal of the sport now that death and injury are unlikely events (13). In our opinion, removing these threats allows driver skill and athleticism to take center stage, possibly shifting success away from those with hubris toward those with expertise and talent.

### 3.3 Stigma and gender issues

At the time of writing, all competing elite F1 drivers are male and gender-specific inquiries remain negligible (1, 2) While there has recently been increasing openness towards mental health in F1, stigma and outdated sociocultural stereotypes still endure (96). Not every driver has embraced open mental health discourse, with some reaffirming harmful conceptions or stigma and conflicting mindsets about F1's core identity (97). When asked if he would be open to discussing mental illness, the current F1 champion, Max Verstappen, noted: “Even if you have one, I would never say so […] why would you say you’re weak even if you have one” (98). These enduring attitudes resemble those identified in other sporting domains, where mental health stigma remains a persistent barrier for engagement and advances in sports psychiatry (24).

Elsewhere, reasons for the limited female representation have been discussed (99). Nonetheless, motorsport science literature only includes negligible samples of female drivers, primarily in amateur circuits with a mix of open and closed-cockpit vehicles (100). It is important to contextualize this lack of gender-specific information with the positive and concerted efforts to encourage female F1 participation and the psychosocial challenges that other elite female athletes have faced when entering traditionally male-dominated sports (101, 102). As of 2023, women are being proactively recruited and trained in the F1 Academy program, though drivers have already discussed their experiences with mental health challenges, some of which are pre-existing, but others may have arisen due to racing pressures (103, 104). Interestingly, the intra-race environment has been the sole area of study involving female athletes, with researchers finding no gender-based difference in heart rate (100). This outcome serves to refute ideas that female physiology would negatively impinge upon competitive ability, as is demonstrated by women's success in other motorsports, such as the Indy 500 (105). However, this has not influenced certain perceptions around the ability of female drivers to compete (106).

In conjunction with this, scholars have illustrated the persistence of outdated stereotypes around F1. These include the “Playboy” characteristics of drivers and the overall bravado of risky behaviors (96). Discussing his mental health symptoms, Jenson Button, underlined the prevalence of stereotypically “macho” aspects that contributed to his depression and anxiety (5). Moreover, from a gender-based perspective, it is likely that this has a distinct impact on female drivers as they attempt to rise in the ranks, and women have described their experiences with sexism and discrimination (18, 107). More broadly, the outdated message that F1 is only for men may have discouraged female participation (18). Thus, notwithstanding progressive efforts to bolster female engagement, complex and outdated questions still arise from certain commentators about whether women are physically and mentally capable to drive and the implications for men when they do (18).

## 4 Recommendations for promoting mental wellbeing across F1

### 4.1 Research and mental health programs for F1 drivers

Due to the guarded nature of F1 and the intensity of competitive racing, there may be limited opportunities to conduct robust academic research on mental health. Notably, *N* = 20 current active drivers compete on the Grand Prix circuit, with approximately *N* = 30–50 reserve/third drivers per season. High sensitivities from teams and stakeholders and concerns around intellectual property inevitably renders the scope of certain scholarly studies unfeasible (108, 109).

Despite the paucity of evidence and shifting priorities within F1 management, there is a growing acknowledgment of the necessity for a paradigm shift, demonstrated by increased efforts to raise mental health awareness. This is exemplified by initiatives like the “We Race as One” campaign (110). While mental health stigma still endures (98), the F1 organization and several teams have repeatedly used World Mental Health Day (October 10) to underscore the challenges experienced by the drivers, managers, and staff members (111, 112).

In sporting contexts, investigations into athlete psychology are often framed around performance; nevertheless, it is essential that mental wellbeing is separated from these objectives to support sustainable health outcomes (113). Instead, sufficient psychiatric research, assessment, and treatment for mental disorders in F1 should be emphasized, as in other sports, irrespective of performance-based goals (23). Team transparency may be a key facilitator, allowing for greater scrutiny by sports psychiatrists and allied health professionals to strengthen available mental health schemes. In this regard, many teams have likely invested in mental health care, but it remains difficult to scrutinize these protocols and determine whether they are based on up-to-date scientific evidence and best practice (114). Accordingly, a standardized protocol may benefit both current F1 drivers and younger drivers within the driving academies and junior circuits. To that end, previous initiatives across other sports and elite athlete groups have been developed [e.g., (25)] and more robust interventions could be adapted and refined for the specificities of F1.

Yet, across different sports, there has been a general reluctance among certain athletes to engage in mental health assessment and significant help-seeking barriers exist [e.g., (114, 115)], though drivers have discussed their positive experiences in seeking mental health support (64, 113). Whilst certain health and safety measures in F1 had been met with initial discontent, benefits continue to accrue (116), yet negative perceptions about mental health likely remain a sizable obstacle (24, 117). Ultimately, even rigorous assessment regimes may be impaired if they are deemed to confirm stereotypes or undermine performance (24). As prevention of mental disorder is predicated on the ability to assess the individual, this lack of understanding may mean that drivers continually pursue curative measures or maladaptive coping mechanisms that could disrupt training, racing, and other responsibilities. Hence, to maximize the success of mental health protocols, it should be mandatory that all teams and drivers receive education on various mental health topics, with subsequent follow-up and monitoring (where applicable).

### 4.2 Regulatory actions

Previously, the FIA have also enforced other progressive changes to respond to driver-centric concerns, such as amending body weight regulations (46). Additional adjustments could be made to promote the driver mental wellbeing. As the Grand Prix schedule has created difficulties in physiological responses to travel as well as in logistical planning for both drivers and teams, efficient redesign of travel plans could ameliorate psychological stressors (44). Furthermore, media and sponsor obligations should be examined and subsequently prioritized by necessity with clear opt-out options. We acknowledge that the presence of the drivers at these events is integral to enhancing the image of the sport and sponsor value (118). However, it is important to remember that unlike the vehicles they drive, drivers require adequate rest and recovery.

The FIA has taken steps to promote a positive sporting environment and prioritize mental health (119). We applaud the introduction of these policies and strongly advocate for safeguarding measures to be implemented that safeguard the mental health of all drivers across their careers, beginning in the amateur race circuits and driver academies. Given evidence suggesting that young children, adolescents, and drivers at the end of their career may face adverse experiences, relevant programs should encompass sufficient protection for potentially vulnerable groups. Educational efforts could be implemented to help young athletes remain passionate and motivated without exhibiting adverse behaviors or frustration (76). We also support actions like limiting the number of hours that children and younger athletes participate in training exercises or implementing awareness programs for physical and sexual abuse, as discussed elsewhere (120). As research has shown that coping tactics for retirement can help athletes manage identity concerns, we advocate for counseling and support for athletes at the end of their driving career as well (121), again promoting the notion of sustainable wellbeing across the lifespan (4).

Since more women are encouraged to enter F1, there have been proposals to introduce wider safeguarding measures (e.g., for the behavior of race marshalls) (122). Likewise, welcome changes have been made to traditional and regressive aspects of Grand Prix pageantry, such as the elimination of “Grid Girls” in 2018 (123). Therefore, regulatory actions are necessary for creating an environment that supports diversity and inclusion, which should be continued and expanded by the FIA as F1 grows in influence and popularity.

### 4.3 Media presentations, advocacy, and mentorship

As media attention in F1 expands, we hope that open dialogues about mental health become more normalized. In other sports, media depictions that portray athletes as human beings with emotions and vulnerabilities have served to increase safety and support systems (124). Stakeholders, including sponsors, should be encouraged to advance the idea that drivers are athletes, not entertainers, and more realistic representations of racing on the Grand Prix circuit could help ameliorate this. In turn, this can influence public attitudes and research agendas to focus more on the athletic characteristics of F1 drivers and motorsport participants in general.

Together with this, as they have highlighted, drivers themselves have a unique opportunity to cultivate a culture of wellbeing amongst both current and future athletes (101), which could be facilitated by the Grand Prix Drivers’ Association. Analogously, in sports medicine literature, mentorship programs among elite athletes have been shown to improve confidence, alongside performance for individual athletes (125). Similar schemes should be refined within F1 with the aim of yielding mental health benefits and building resilience. This may be particularly important for post-career transitions, which can present significant social, psychological, economical, and even physical challenges (126). Notably, recent research suggests that F1 driver's career longevity may be declining overall, potentially highlighting an increased need for support (127) and again strengthening the notion of mental wellbeing across all life trajectories.

## 5 Conclusion

F1 is a dynamic and demanding sport, which could conceivably lead to significant physiological and psychological and increased vulnerabilities for mental health issues. Current limitations in the evidence-base around driver science makes it difficult to ascertain general health risks and to speculate on the prevalence of psychiatric symptoms. Likewise, more research is required to predict the success of treatment protocols and clinical outcomes for both physiological and mental illness. With regard to psychiatric etiology, it is also important to understand if athletes who become F1drivers share certain characteristics that render them more susceptible for mental disorder or if sport-specific factors contribute to psychopathology. Integrating these research questions and sports psychiatry approaches into the wider area of motorsport science could be vital for supporting advancements in mental health support in F1. Despite the lack of data, there have been numerous insights from drivers across the sport about the mental health challenges and the risk factors inherent within F1, which intersect with those observed in different sporting domains. In our view, the enhancement of F1 should be closely interrelated with upon increased efforts to ensure driver wellbeing across the lifespan.

Multifaceted approaches to raise mental health awareness and the efforts of drivers to promote open conversations should be applauded. Nonetheless, inquiries into the existence of mental disorder, alongside standardized psychiatric screening, safeguarding measures, and broader changes to the sociocultural perceptions around drivers as entertainers should be encouraged. Increased information sharing and knowledge exchanges can advance these goals, emphasizing the development of targeted and robust mental health initiatives to mitigate potential challenges and improve wellness from the beginning to the end of a driver's career.

## Data Availability

The original contributions presented in the study are included in the article/Supplementary Material, further inquiries can be directed to the corresponding author.
